# APOBEC3G Oligomerization Is Associated with the Inhibition of Both *Alu* and LINE-1 Retrotransposition

**DOI:** 10.1371/journal.pone.0084228

**Published:** 2013-12-19

**Authors:** Takayoshi Koyama, Juan Fernando Arias, Yukie Iwabu, Masaru Yokoyama, Hideaki Fujita, Hironori Sato, Kenzo Tokunaga

**Affiliations:** 1 Department of Pathology, National Institute of Infectious Diseases, Tokyo, Japan; 2 Pathogen Genomics Center, National Institute of Infectious Diseases, Tokyo, Japan; 3 Faculty of Pharmaceutical Sciences, Nagasaki International University, Nagasaki, Japan; Lady Davis Institute for Medical Research, Canada

## Abstract

*Alu* and LINE-1 (L1), which constitute ~11% and ~17% of the human genome, respectively, are transposable non-LTR retroelements. They transpose not only in germ cells but also in somatic cells, occasionally causing cancer. We have previously demonstrated that antiretroviral restriction factors, human APOBEC3 (hA3) proteins (A–H), differentially inhibit L1 retrotransposition. In this present study, we found that hA3 members also restrict *Alu* retrotransposition at differential levels that correlate with those observed previously for L1 inhibition. Through deletion analyses based on the best-characterized hA3 member human APOBEC3G (hA3G), its N-terminal 30 amino acids were required for its inhibitory activity against *Alu* retrotransposition. The inhibitory effect of hA3G on *Alu* retrotransposition was associated with its oligomerization that was affected by the deletion of its N-terminal 30 amino acids. Through structural modeling, the amino acids 24 to 28 of hA3G were predicted to be located at the interface of the dimer. The mutation of these residues resulted in abrogated hA3G oligomerization, and consistently abolished the inhibitory activity of hA3G against *Alu* retrotransposition. Importantly, the anti-L1 activity of hA3G was also associated with hA3G oligomerization. These results suggest that the inhibitory activities of hA3G against *Alu* and L1 retrotransposition might involve a common mechanism.

## Introduction

Retrotransposons compose ~42% of the human genome, and these elements are classified into the non-LTR and LTR classes. Non-LTR retrotransposons are subdivided into long interspersed elements (LINEs) and short interspersed elements (SINEs), representatives of which are LINE-1 (L1) and *Alu*, which comprise ~17% and ~11% of the human genome, respectively [1]. L1 elements harbor two ORFs: ORF1, which encodes an RNA-binding protein, and ORF2, which encodes an endonuclease-like and reverse transcriptase-like protein. After translation, these proteins bind to the L1 RNA to form a ribonucleoprotein particle that is imported into the nucleus to be integrated into the genome through target-primed reverse transcription [2-4]. Unlike L1, *Alu* elements do not encode a reverse transcriptase or an endonuclease; rather, the transcribed *Alu* RNAs hijack the L1-encoded enzymes to move to new locations in the genome through mechanisms that are as yet unclear [5]. Importantly, retrotransposition by L1 and *Alu* occurs not only in germ cells, causing several genetic diseases [6-13], but also in somatic cells, such as brain tissues [14,15], and malignant tissues and cells such as B-cell lymphoma cells [16], breast carcinoma tissue [17], colon carcinoma tissue [18], and hepatocellular carcinoma tissue [19]. These facts indicate that an intrinsic protection system should function properly to suppress these types of retrotransposition in normal somatic cells. 

Human APOBEC3G (hA3G) is one of the seven members of the APOBEC3 (hA3) family of cytidine deaminases (hA3A to hA3H). hA3G is known to be an intrinsic retroviral restriction factor that inhibits Vif-defective human immunodeficiency virus type 1 (HIV-1) infection by being incorporated into viral particles and mediating extensive deamination of the nascent minus-strand viral DNA during reverse transcription, which results in G-to-A hypermutation [20-23]. This antiretroviral restriction extends to not only exogenous retroviruses, such as simian immunodeficiency virus [24-27], primate foamy virus [28,29], human T-cell leukemia virus type I [30], murine leukemia virus [21,26,31], mouse mammary tumor virus [32], and equine infectious anemia virus [22] , but also endogenous retroelements, such as the MusD and intracisternal A-particle LTR murine retrotransposons and, as described below, human *Alu* and L1 retrotransposons ([33-40]; see also review in ref[41].). hA3G also restricts infection by hepatitis B virus, which replicates its DNA genome by reverse transcription of an RNA intermediate [42,43]. Whereas pre-primate mammals encode one, two to three A3 proteins [44], primates have acquired seven different A3 genes through 33 million years of evolution [45]. Such expansion of the hA3 genes correlates with an abrupt reduction in retrotransposition activity in primates, suggesting that these proteins have evolved to protect hosts from the genomic instability caused by retroelements [46].

We previously reported that hA3 family proteins have differential levels of anti-L1 activity that do not correlate with either antiretroviral activity or subcellular localization patterns [37]. Although several groups that performed similar studies showed that hA3G has little or no anti-L1 activity [47-50], we and others have found that the hA3G is indeed able, albeit less potently than hA3A or hA3B, to restrict L1 retrotransposition [37-40]. Such discrepancies might be attributed to the cell-type-dependent expression levels of hA3G, as we previously demonstrated [37]. We also found that hA3G inhibits L1 retrotransposition independently of its deaminase activity, which is primarily required for its antiretroviral function, and hA3G likely prevents L1 DNA synthesis *per se* [37]. With regard to the inhibition of *Alu* by hA3 family members, several groups have reported that hA3A, hA3B [49], hA3G [34-36], hA3DE, and hA3H [51] inhibit *Alu* retrotransposition. In this study, we found that all hA3 family members, from hA3A to hA3H, are able to inhibit *Alu* retrotransposition. The inhibitory effect of hA3G on *Alu* retrotransposon was associated with the N-terminal 30 amino acid residues and with hA3G’s oligomerization activity, but not with its deaminase activity. Structural modeling showed that amino acid positions 24–28 are responsible for the oligomerization of hA3G. This result was verified by immunoprecipitation using an hA3G mutant with amino acid substitutions at these positions. Consistent with this result, we found that amino acid positions 24–28 of hA3G are critical for its inhibitory activity against *Alu* retrotransposon. Importantly, these amino acids were also shown to be important for L1 inhibition, suggesting that both *Alu* and L1 retrotransposition might be restricted by similar mechanisms involving hA3G, which require the oligomerization of this restriction factor.

## Materials and Methods

### DNA constructs

The hemagglutinin (HA)-tagged hA3 expression plasmids (phA3A-HA, phA3B-HA, phA3C-HA, phA3DE-HA, phA3F-HA, phA3G-HA, and phA3H-HA), the GFP expression plasmid pCA-EGFP, the empty expression vector pCAGGS-HA, the L1 indicator construct pCEP4/L1mneoI/ColE1 (kindly provided by N. Gilbert), the L1 ORF2 expression plasmid pBudORF2opt (kindly provided by A.M. Roy-Engel), the *Alu* indicator construct pYa5neotet (kindly provided by T. Heidmann), Vif-deficient HIV-1 proviral indicator construct pNLLuc-F(-)E(-), and VSV-G expression plasmid pHIT/G have previously been described elsewhere [5,37,52-55] (note that the hA3h expression plasmid encodes the haplotype I). The myc-tagged version of the wild-type hA3G expression plasmid, phA3G-myc, was also created. A series of N-terminal deletion mutants of hA3G (phA3G-NΔ30-HA, -NΔ60-HA, -NΔ90-HA, -NΔ120-HA, and -NΔ150-HA) were created by inserting serially deleted PCR fragments of hA3G into the mammalian expression plasmid pCAGGS with a C-terminal HA-tag. The deaminase-deficient mutant (phA3G-E259Q-HA), the oligomerization-deficient mutant (phA3G-C97/100A-HA), and the N-terminal mutants (phA3G-5G(24–28)-HA, phA3G-4G(124–127)-HA, phA3G-R24G-HA, and phA3G-Y125G-HA) of hA3G were created using phA3G-HA as a template with a QuikChange site-directed mutagenesis kit (Stratagene).

### Cell maintenance, transfections, and protein analyses

HeLa and 293T cells were maintained under standard conditions. 293T cells were transfected with HA-tagged hA3 wild-type and mutant plasmids using the FuGENE 6 transfection reagent (Roche Applied Science) according to the manufacturer’s instructions. Cell extracts from transfected cells were subjected to gel electrophoresis and then transferred to a nitrocellulose membrane. The membranes were probed with an anti-HA mouse monoclonal antibody (Sigma). The antibody-bound proteins were visualized to confirm hA3 protein expression by chemiluminescence using an ECL Western blotting detection system (GE Healthcare) and an LAS-3000 imaging system (FujiFilm).

### Immunofluorescence microscopy

HeLa cells were plated on 13-mm glass coverslips and transfected with 0.5 µg of hA3 expressing plasmids by using FUGENE6. The transfected cells were fixed with 4% paraformaldehyde at room temperature for 30 min, permeabilized with 0.05% saponin for 10 min, and immunostained with an anti-HA monoclonal antibody (5 µg/ml). The secondary goat anti-mouse antibody that was conjugated with Cy3 was used at 5 µg/ml. All immunofluorescence images were observed on a Leica DMRB microscope (Wetzlar, Germany) equipped with a 63×1.32 NA oil immersion lens (PL APO), acquired through a cooled CCD camera, MicroMAX (Princeton Instruments, Trenton, NJ), and digitally processed using IPlab Software (Scanalytics, Fairfax, VA). All images were assembled using Adobe Photoshop (Adobe Systems, Mountain View, CA).

### L1 and Alu retrotransposition assay

L1 and *Alu* retrotransposition assays were performed by co-transfecting 2 x 10^5^ HeLa cells with 0.1 μg of the respective hA3 expression plasmid (or a mock expression vector, pCAGGS-HA, as a positive control) together with either 0.3 μg of the neomycin-resistance (neo^r^)-based L1 expression vector pCEP4/L1mneoI/ColE1 and 0.1 μg of an empty vector (for the L1 retrotransposition assay) or 0.3 μg of the neo^r^-based *Alu* expression vector pYa5neotet and 0.1 μg of the L1 ORF2 expression plasmid pBudORF2opt (for the *Alu* retrotransposition assay) using Lipofectamine and Plus reagents (Invitrogen). As a negative control, 0.5 μg of a GFP expression vector, pCA-EGFP, was transfected into HeLa cells. After 72 h, the cells were trypsinized, re-seeded into T25 or T75 flasks for G418 selection (1 mg/ml for the L1 assay and 400 μg/ml for the *Alu* assay), and maintained. At 14 days after selection, the resultant G418-resistant (G418^R^) colonies were fixed, stained with crystal violet (Merck), and counted.

### Oligomerization assay

To perform a coimmunoprecipitation-based oligomerization assay, plasmids (0.5 μg) expressing HA-tagged wild-type and mutant hA3G were transfected along with phA3G-myc (0.5 μg) into 293T cells using FuGENE 6. After 48 h, the transfected cells were suspended in 500 μl of RIPA buffer (50 mM Tris-HCl, pH 7.4, 150 mM NaCl, 1% NP-40, 0.5% sodium deoxycholate, 0.1% SDS, complete protease inhibitor cocktail [Roche]). The resultant lysates were clarified by brief centrifugation, pre-cleared with 30 μl of Protein A-Agarose Fast Flow (GE Healthcare) for 1 h at 4°C, and then incubated with an anti-myc affinity gel (Sigma). After 1 h at 4°C, the immune complexes were extensively washed with RIPA buffer. Equal aliquots of the total and bound fractions were subjected to gel electrophoresis and transferred to a nitrocellulose membrane. The membranes were probed with an anti-HA mouse monoclonal antibody (Sigma) or an anti-β-actin mouse monoclonal antibody (AC-74, Sigma). The signal intensities of the immunoprecipitated hA3G protein on Western blots were quantified using the LAS-3000 imaging system (Fujifilm). For the RNase A treatment experiment, the immune complexes were separated into two aliquots. The wild-type sample was incubated with or without 25 U of RNase A (Sigma) at room temperature for 30 min. Samples were extensively washed and then resuspended in loading dye. The samples were assayed as described above.

### Molecular modeling of the head-to-head dimer structure of the N-terminus of hA3G

Head-to-head dimer models of hA3G N-terminal domain were obtained by homology modelling using either the crystal structure of human APOBEC2 (hA2) at a resolution of 2.50 Å or the NMR structure of the C-terminal domain of hA3G (PDB code: 2NYT chain A [56] or 2JYW [57], respectively) as a template, as previously performed [34,58-60]. To minimize misalignments between the hA3G N-terminal domain as a target sequence and either hA2 or the C-terminal domain of hA3G as a template sequence, we used the multiple sequence alignment method with the sequences of hA3A (GenBank accession number: NM_145699), hA3C (GenBank accession number: NM_014508), and hA3F (GenBank accession number: NM_145298). Multiple sequence alignments were generated using ‘MOE-Align’ in Molecular Operating Environment (MOE) version 2010.10 (Chemical Computing Group Inc., Quebec, Canada). Three-dimensional (3-D) models of the hA3G N-terminal domain were constructed by the homology modeling technique using ‘MOE-Homology’ in MOE as previously described [61]. We obtained 25 intermediate models per homology modeling session in MOE, and we selected the 3-D models that were intermediate models with best scores according to the generalized Born/volume integral methodology [62]. The 3-D structure was thermodynamically optimized by energy minimization using MOE and an AMBER99 force field [63] combined with the generalized Born model of aqueous solvation implemented in MOE [64]. The physically unacceptable local structure of the optimized 3-D model was further refined based on the evaluation of the Ramachandran plot using MOE. 

## Results

### hA3 family members differentially inhibit Alu retrotransposition

**Figure 1:**
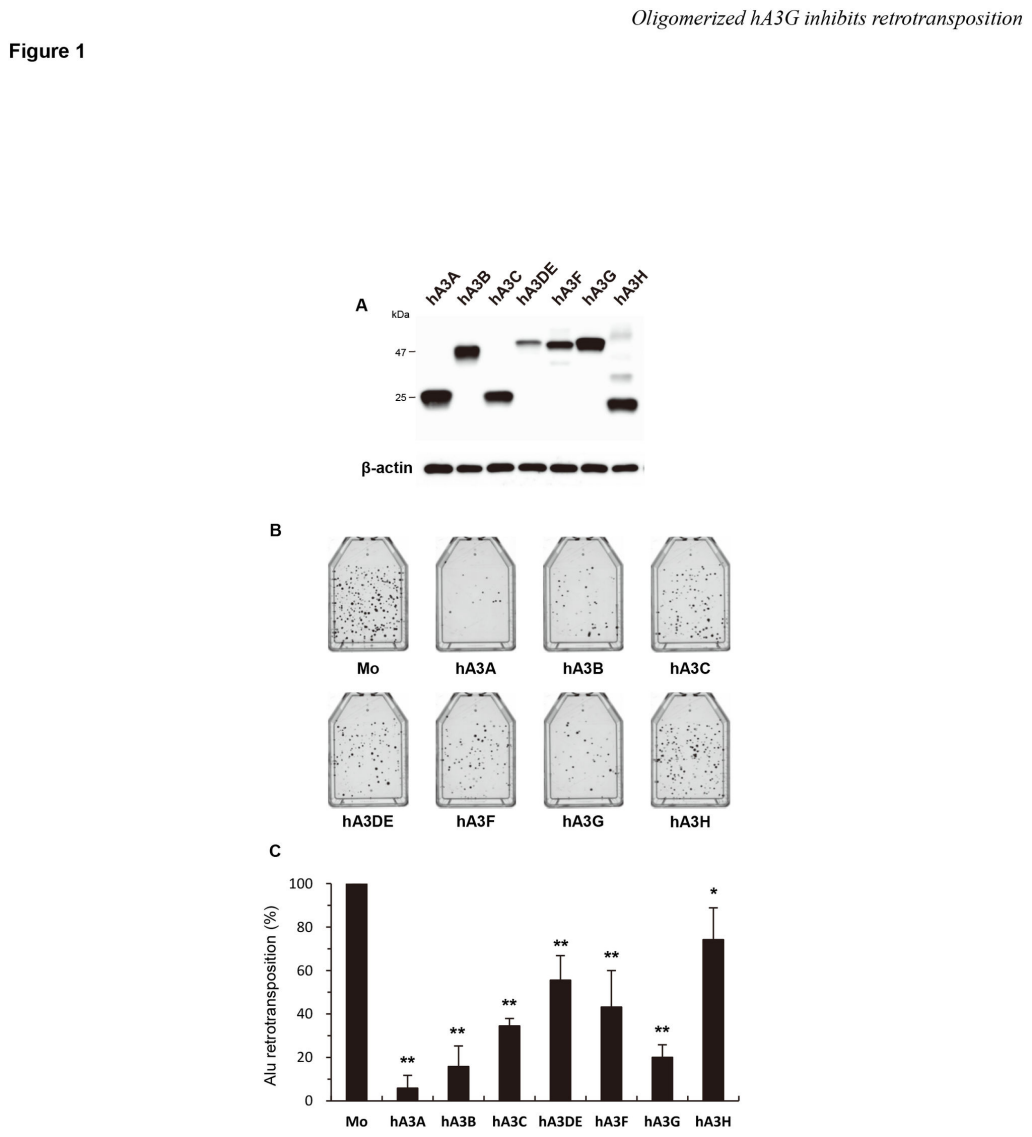
hA3 proteins inhibit Alu retrotransposition at differential levels. (**A**) Western blot analysis was performed by using extracts from 293T cells transfected with HA-tagged hA3 expression plasmids. Antibodies specific for HA were used. (**B**, **C**) HeLa cells were cotransfected with the *neo* ^*r*^-based *Alu* expression vector pYa5neotet and the L1 ORF2 expression plasmid pBudORF2opt, together with the respective hA3 expression plasmid. Seventy-two hours later, the cells were trypsinized, re-seeded into T25 or T75 flasks, and subjected to G418 (400 μg/ml) selection. At 14 days after selection, the resultant G418^R^ colonies fixed, stained with crystal violet (B), and counted to determine the level of *Alu* retrotransposition (C). The retrotransposition level in the absence of hA3 proteins was set to 100%. The data shown are the mean ± SD of triplicate experiments. Mo, mock. *P < 0.05, **P < 0.005, *t*-test.

To determine if hA3 family members are able to inhibit *Alu* retrotransposition as well as L1 retrotransposition [37], we performed a neo^r^-based retrotransposition assay [5]. In this assay system, we utilized a L1 ORF2 expression plasmid that is required for *Alu* retrotransposition [53], together with an *Alu* clone DNA carrying a reverse-oriented neo^r^ gene separated by a gamma-globin intron. After transfection of the cells with this construct, neo^r^ with *Alu* is transcribed, spliced, reverse-transcribed, and integrated. Then, the neo^r^ gene that is integrated with *Alu* is driven by the CMV promoter and expressed. After G418 selection following transfection, we were able to quantify the retrotransposition level by counting the number of G418^R^ colonies. hA3 protein expression in the cells were confirmed by immunoblotting using anti-HA antibodies (Figure 1A). Without the co-expression of an hA3 protein, *Alu* retrotransposition occurred at the level shown in the upper left panel of Figure 1B. In contrast, the co-expression of any of the hA3 proteins differentially inhibited *Alu* retrotransposition, and in particular, the expression of hA3A, hA3B, or hA3G strongly decreased the transposition level of *Alu* elements (Figure 1C). Thus, we conclude that hA3 proteins act to differentially suppress *Alu* retrotransposition. Importantly, in agreement with previous reports [34-36,39], we observed that hA3G has an inhibitory effect on *Alu* retrotransposition in the assay. It should be noted that these activities against *Alu* correlated exactly with the patterns previously observed for the inhibition of L1 [37].

### The *N-*terminal 30 amino acids of hA3G determine the inhibitory effect on Alu retrotransposition

**Figure 2:**
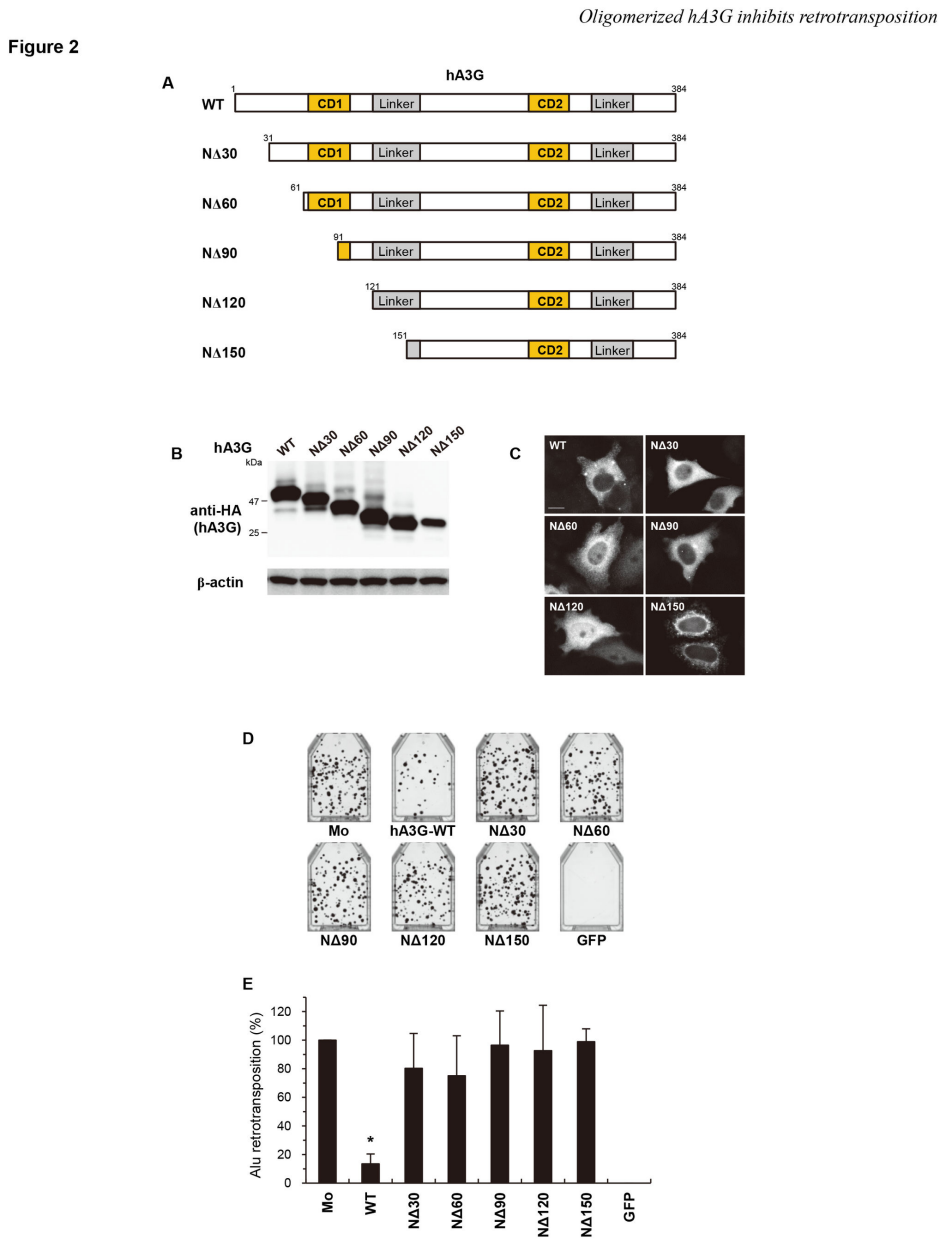
The N-terminal 30 amino acids regulate the anti-Alu activity of hA3G. (**A**) Schematic depiction of a series of N-terminal deletion mutants of hA3G. CD1, N-terminal cytidine deaminase; CD2, C-terminal cytidine deaminase. (**B**) Western blot analysis was performed using extracts from 293T cells transfected with plasmids expressing HA-tagged hA3G mutant proteins. Monoclonal antibodies specific for HA (upper) or β-actin (lower) were used. (**C**) Representative images of HeLa cells transfected with the indicated plasmids are shown. hA3G wild-type (WT), NΔ30, NΔ60, NΔ90, and NΔ120 mutant proteins were predominantly localized to the cytoplasm, whereas NΔ150 mutant protein localized to the perinuclear region. Scale bar: 20 µm. (**D**, **E**) An *Alu* retrotransposition assay was performed as described in Figure 1. Crystal violet-stained G418^R^ colonies were counted to determine the level of *Alu* retrotransposition. The data shown are the mean ± SD of triplicate experiments. Mo, mock; WT, wild-type hA3G; GFP, GFP only. *P < 0.005, *t*-test.

Because hA3G is the best characterized hA3 family member protein, we focused on this protein and attempted to determine the region responsible for its anti-retrotransposon activities. To identify the relevant region, we created a series of mutants with serial deletions from the N-terminus up to amino residue 150 (Figure 2A). Protein expression in the cells transfected with each plasmid was confirmed by immunoblotting using an anti-HA antibody (Figure 2B). hA3G mutants lacking the C-terminal domain were undetectable as previously reported [34,65] and therefore could not be used for further experiments. Immunofluorescence microscopy confirmed that hA3G deletion mutant proteins other than NΔ150 were predominantly localized to the cytoplasm, as was the wild-type protein (Figure 2C). These deletions also abrogated the anti-HIV-1 activity of hA3G (Figure S1). We performed an *Alu* retrotransposition assay by transfecting HeLa cells with the *Alu* expression plasmid, the L1 expression plasmid, and a wild-type or mutant hA3G plasmid, and we observed that the deletion of 30 or more residues from the N-terminus of hA3G completely abrogated the inhibitory activity of hA3G on *Alu* retrotransposition (Figures 2D and 2E). We therefore conclude that the N-terminal 30 amino acids of hA3G are critical for the inhibition of *Alu* retrotransposition.

### The inhibitory effect of hA3G on Alu retrotransposition is associated with its oligomerization and is independent of its deaminase activity

**Figure 3:**
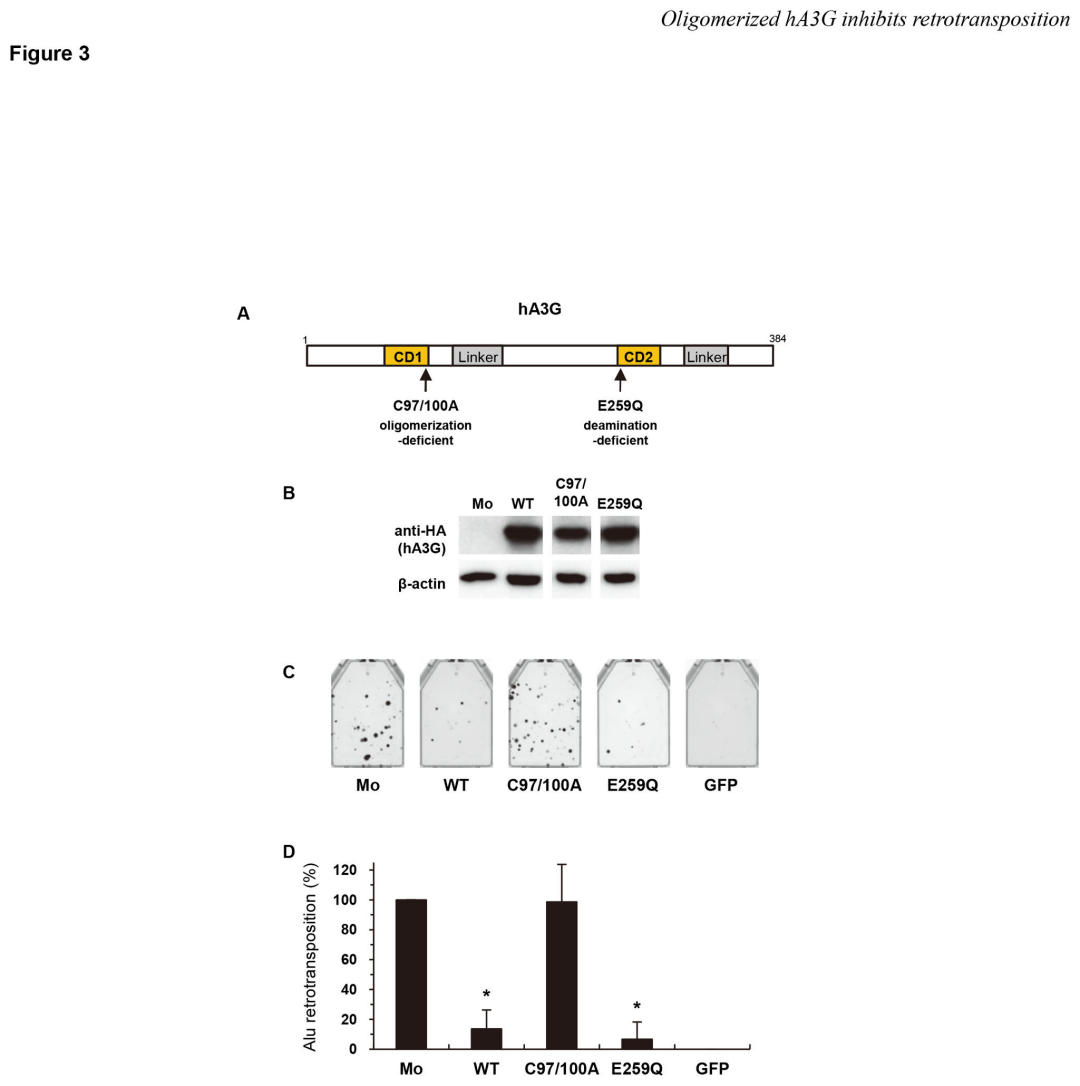
The anti-Alu activity of hA3G is associated with its oligomerization and is independent of its deaminase activity. (**A**) Schematic depiction of two mutants: an oligomerization-deficient mutant, C97/100A, and a deamination-deficient mutant, E259Q. (**B**) Western blot analysis was performed using extracts from 293T cells transfected with plasmids expressing HA-tagged hA3G mutant proteins. Monoclonal antibodies specific for HA (upper) or β-actin (lower) were used. (**C**, **D**) An *Alu* retrotransposition assay was performed as described in Figure 1. A GFP expression vector was used as a negative control. Crystal violet-stained G418^R^ colonies were counted to determine the level of *Alu* retrotransposition. The data shown are the mean ± SD of triplicate experiments. Mo, mock; WT, wild-type hA3G; GFP, GFP only. *P < 0.005, *t*-test.

The anti-HIV-1 activity of hA3G is known to be dependent on two different activities, deamination and oligomerization, the former of which has been shown to be disrupted by the mutation of E259 located in the C-terminal cytidine deaminase (CD2) [65,66], and the latter of which has been reported to be abrogated by the mutation of C97 and C100 located in the N-terminal cytidine deaminase (CD1) [65]. Based on these past findings, we wished to determine which functions of hA3G are crucial for blocking the ability of *Alu* to retrotranspose. We created plasmids expressing hA3G defective in either oligomerization or deamination (C97/100A or E259Q, respectively; Figure 3A) and confirmed the expression of these proteins by immunoblotting using an anti-HA antibody (Figure 3B). Interestingly, the *Alu* retrotransposition assay revealed that the C97/100A oligomerization mutant of hA3G had no inhibitory activity against *Alu* retrotransposition, whereas the E259Q deamination mutant retained wild-type activity (Figure 3C). These observations confirmed the previous results [34,35], showing that the inhibition of *Alu* retrotransposition by hA3G is not due to the ability of hA3G to deaminate this retrotransposon but is due to its ability to form an oligomer. 

### The *N-*terminal 30 amino acids of hA3G are required for the oligomerization of this protein

**Figure 4:**
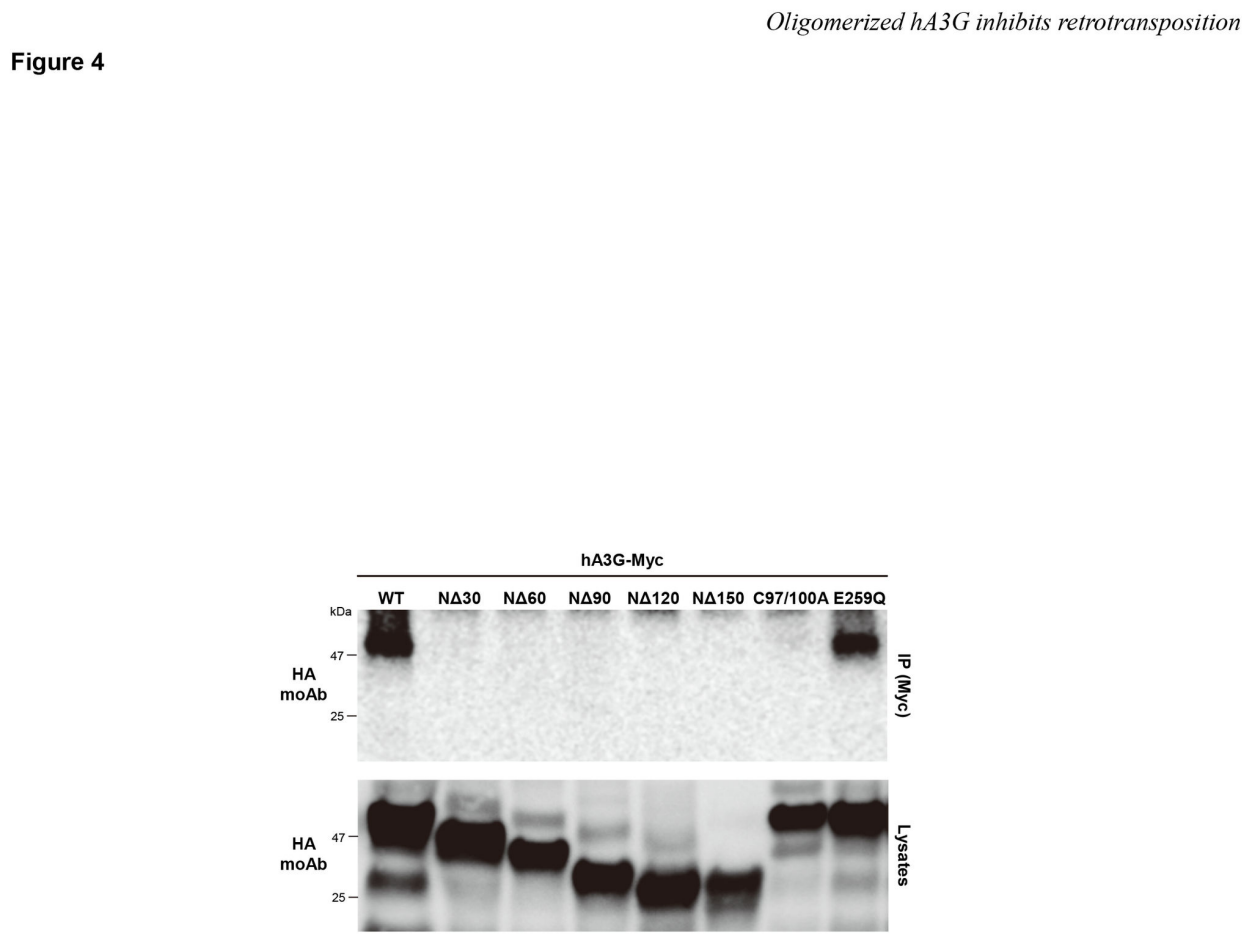
The homooligomerization of hA3G is dependent on the N-terminal 30 amino acid residues of this protein. 293T cells cotransfected with the Myc-tagged and HA-tagged hA3G expression plasmids were immunoprecipitated (IP) with an anti-Myc polyclonal antibody. The resulting complexes were analyzed by immunoblotting with a monoclonal antibody against the HA tag to detect oligomerized hA3G (upper). Cell lysate aliquots were also analyzed in parallel by immunoblotting for the HA tag (lower). WT, wild-type hA3G.

Because hA3G’s inhibitory activity against *Alu* retrotransposition was abolished in the mutants carrying an N-terminal deletion of 30 or more residues (Figure 2) and in the oligomerization mutant harboring mutations at amino acid positions 97 and 100 (Figure 3), we reasoned that the N-terminal 30 amino acids of hA3G might be critical for its ability to form oligomers. To test this hypothesis, we performed an oligomerization assay by coexpressing wild-type hA3G tagged with Myc and the mutant hA3Gs tagged with HA. The cell lysates were then immunoprecipitated with an anti-HA antibody and immunoblotted with an anti-Myc antibody. As shown in Figure 4, the E259Q deamination mutant of myc-tagged hA3G was efficiently coimmunoprecipitated with the HA-tagged wild-type protein. In contrast, the N-terminal serial deletion mutants lacking 30 or more residues completely lost the ability to oligomerize, as did the C97/100A mutant. When the immunoprecipitated samples were treated with RNase A, the oligomerization efficiency of hA3G was moderately decreased (Figure S2), consistent with the previous reports that cellular RNA might contribute to the stabilization of hA3G’s oligomer [34]. Thus, the 30 amino acids at the N-terminus of hA3G are responsible for its oligomerization. 

### The *N-*terminal 30 amino acids of hA3G are the structural key for its oligomerization

**Figure 5:**
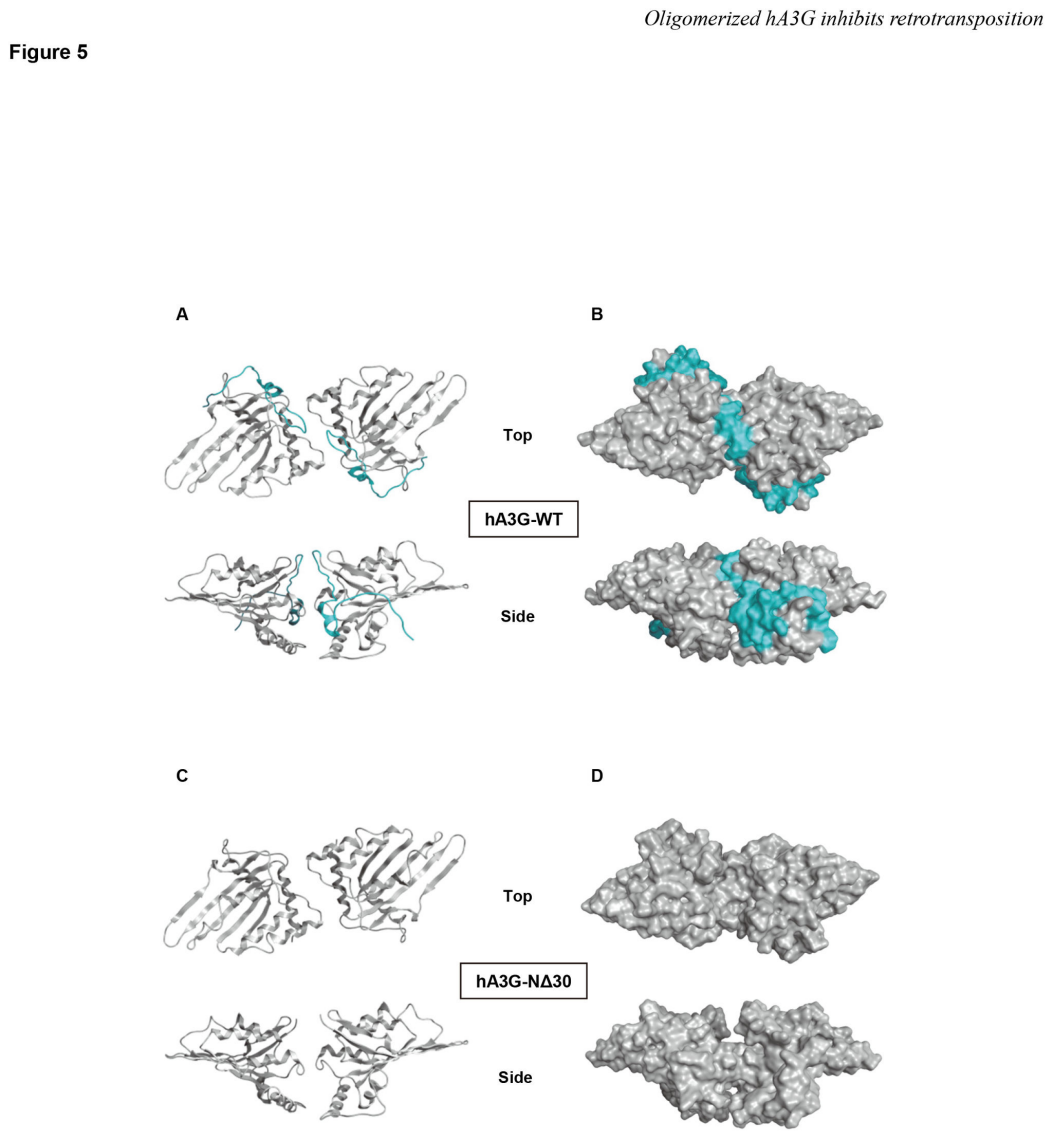
The N-terminal 30 amino acids of hA3G are located at the dimer interface and are therefore key residues for the oligomerization of hA3G. Structural models of the hA3G N-terminal domain. The models were constructed by homology modeling using the X-ray crystal structure of hA2. The head-to-head dimer structure of hA3G N-terminal domain is represented by ribbon models (A and C) and space-filling models (B and D). (**A**, **B**) Views of the top (upper) and side (lower) of wild-type (WT) hA3G. Cyan, N-terminal 30 amino acids of hA3G. (**C**, **D**) Views of the top (upper) and side (lower) of the N-terminal 30-amino-acid deletion mutant of hA3G.

To fully understand the mechanism by which N-terminal 30 amino acids of hA3G regulate oligomerization, we analyzed the effect of the deletion of the N-terminal 30 amino acids on the predicted 3-D structure of the hA3G dimer that was reported to be the major form of hA3G oligomer [34,59]. Thermodynamically stable N-terminal structures of wild-type hA3G and its N-terminal 30-amino-acid deletion mutant were constructed by homology modeling using the hA2 crystal structure as a template. As shown in Figures 5A and B, when the structures of the wild-type and deletion mutant hA3G proteins were compared, it was obvious that the N-terminal 30 amino acids (shown in cyan in Figure 5A) were present along the contact surface of the hA3G dimer, and therefore, the deletion of this region could abolish the interaction interface between the two hA3G molecules. We thus conclude that the N-terminal 30 amino acid residues of hA3G are located at the dimer interface and are critical for oligomerization.

### Residues 24–28 contribute to the ability of hA3G to homooligomerize and inhibit Alu retrotransposition

**Figure 6:**
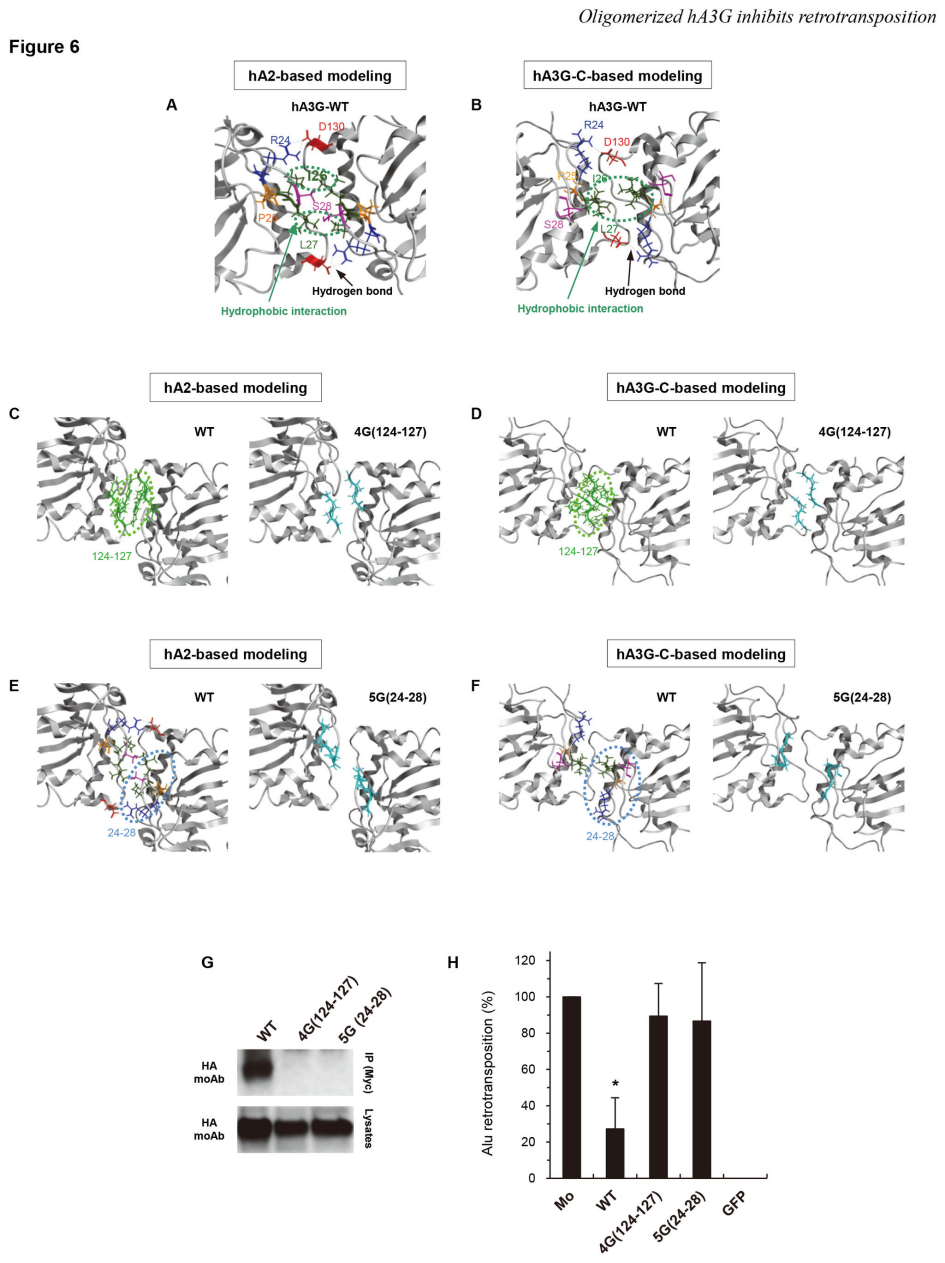
Residues 24–28, as well as known residues 124–127, contribute to the ability of hA3G to homooligomerize and its inhibitory activity against Alu retrotransposition. (A–F) Structural models of hA3G dimer based on the human APOBEC2 (hA2) crystal structure (A, C, and E) and the C-terminal hA3G (hA3G-C) NMR structure (B, D, and F). (**A**, **B**) The interaction surface of the hA3G N-terminal domain in the head-to-head dimer is shown. The hydrophobic interactions formed between either I26 or L27 (green) and their counterpart residues of another monomer (green) are encircled by green dotted lines. A hydrogen bond is formed between a basic residue R24 (blue) and another monomer’s D130 (red). Another hydrogen bond is formed between the S28 residues (pink) of two monomers. Structural stability may be conferred by P25 (orange). (**C**, **D**) The dimer interface at amino acid residues 124–127. Left panel, the aromatic amino acid cluster (YYFW) at positions 124–127 is depicted in light green; right panel, the substitution of these residues with glycines is shown in cyan. (**E**, **F**) The dimer interface at amino acid residues 24–28. Left panel, the dimer interface residues (RPILS) at positions 24–28 are depicted in colors similar to those in A; right panel, substitution of these residues with glycines is shown in cyan. (**G**) IP-Western blot analysis was performed as described in Figure 4; upper, IP; lower, cell lysates. (**H**) An *Alu* retrotransposition assay was performed as described in Figure 1. Crystal violet-stained G418R colonies were counted to determine the level of *Alu* retrotransposition. The data shown are the mean ±SD of triplicate experiments. Mo, mock; WT, wild-type hA3G; GFP, GFP only. *P < 0.05, **P < 0.005, t-test.

Next, we analyzed the interaction interface of the hA3G dimer by structural modeling based not only on the hA2 crystal structure but also on the C-terminal hA3G (hA3G-C) NMR structure in parallel. Both structural modeling of wild-type hA3G revealed that, among the N-terminal 30 amino acids, a cluster of dimer interface residues (R24, P25, I26, L27, and S28) located in the N-terminal core structure α1-loop-β1 of hA3G interact with the counterpart residues of another monomer (Figures 6A and 6B). Importantly, this interface corresponds structurally (but not genetically) to a part of the potential oligomerization interfaces of the hA3G C-terminal domain, as described by Shandilya et al. [67]. At this putative interaction surface (Figures 6A and 6B), R24 likely interacts with D130 of another monomer through hydrogen bonds and electrostatic interactions, whereas the isoleucine/leucine residues at positions 26/27 can form a hydrophobic interaction with the counterpart residues of another monomer. hA2-based modeling shows that the serine residue at position 28 forms another hydrogen bond with the counterpart residues of another monomer (Figure 6A), although the same residue in hA3G-C-based modeling appears to be slightly separated from the counterpart residue of another monomer (Figure 6B). Additionally, the structural stability would be enhanced by a proline residue at position 25 in the loop. Thus, we speculated that the mutation of these residues might abolish the oligomerization of hA3G. To test this hypothesis, we first addressed whether structural modeling would be able to distinguish oligomerization-deficient and oligomerization-intact hA3Gs by analyzing the model of an hA3G mutant (hA3G-4G(124–127)), in which we introduced the small amino acid glycine in place of the aromatic amino acid residues (Y124, Y125, F126, and W127) that have been predicted to be hot spots of protein–protein interaction [68] and have been reported to be critical for the RNA-mediated oligomerization of hA3G [34,59]. The structural model of hA3G-4G(124–127) indicates that the mutant does not form the aromatic cluster, leaving a prominent space between two monomers (compare the left and right panels of Figure 6C and the left and right panels of Figure 6D). Given this result, we then introduced in silico mutations into the five-amino-acid cluster (R24, P25, I26, L27, and S28) of the putative dimer interface (RPILS –> GGGGG; designated 5G(24–28)) (compare the left and right panels of Figure 6E and the left and right panels of Figure 6F). The space between the two monomers of the mutant was clearly comparable to that of 4G(124–127), implying that the 5G(24–28) mutant cannot form a dimer. Based on the structural models, we constructed the myc-tagged N-terminal mutants hA3G-5G(24–28) and hA3G-4G(124–127) to determine whether the former hA3G mutant is unable to physically oligomerize. To assess oligomerization, we performed coimmunoprecipitation-based oligomerization assays using wild-type and mutant hA3G proteins. The 5G(24–28) mutant was not coimmunoprecipitated (Figure 6G), nor was 4G(124–127), suggesting that these mutants do not have the ability to oligomerize. Finally, to determine whether the 4G(124–127) and 5G(24–28) mutants lacks anti-*Alu* activity, we performed the retrotransposition assay and found out that these mutants had completely lost the ability to inhibit *Alu* retrotransposition (Figure 6H). hA3G mutants harboring individual amino acid substitutions (R24G and Y125G) displayed equivalent or moderately less inhibitory activity with comparable dimerization (Figures S3A and S3B). In addition, 5G(24–28) and 4G(124–127) mutations both negatively affect the ability of hA3G to inhibit HIV-1 infection (Figure S4). Taken altogether, these results indicate that the N-terminal amino acid residues 24–28 (RPILS) contribute to the oligomerization of hA3G and its anti-*Alu* retrotransposition activity. 

### hA3G oligomerization is associated with the inhibition of L1 retrotransposition

**Figure 7:**
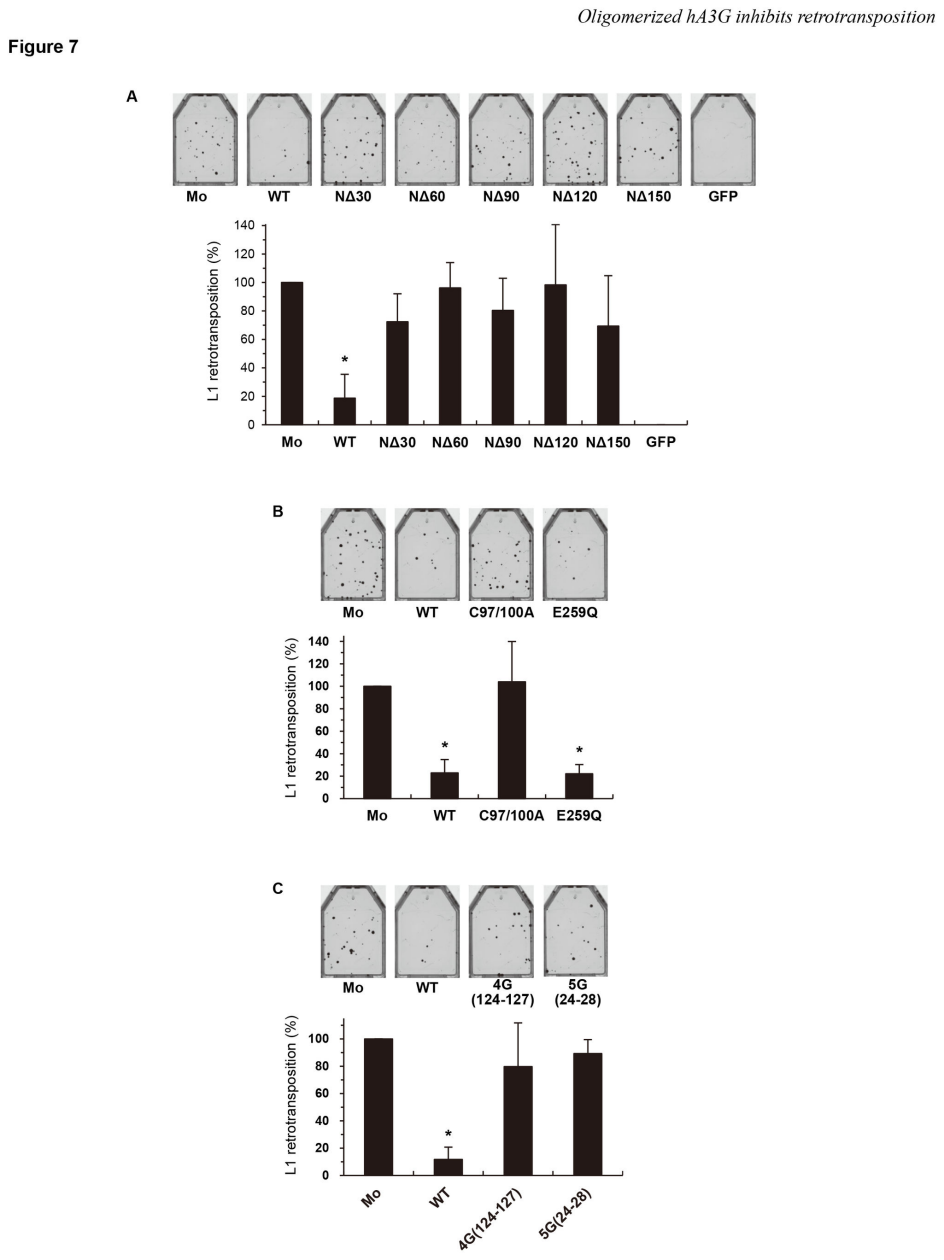
The oligomerization of hA3G is also associated with its anti-L1 activity. HeLa cells were cotransfected with the *neo* ^*r*^-based L1 expression vector pCEP4/L1mneoI/ColE1 and either a wild-type (WT) or mutant hA3G expression plasmids. Seventy-two hours later, cells were trypsinized, re-seeded into T25 or T75 flasks, and subjected to G418 (1 mg/ml) selection. At 14 days after selection, the resultant G418^R^ colonies fixed, stained with crystal violet, and counted to determine the level of L1 retrotransposition. (**A**) Compare the results with Figure 2D and 2E. (**B**) Compare the results with Figure 3C and 3D. (**C**) Compare the results with Figure 6H. The data shown are the mean ± SD of triplicate experiments. Mo, mock; WT, wild-type hA3G; GFP, GFP only. *P < 0.005, *t*-test.

The inhibitory effects of the hA3G protein on *Alu* retrotransposition resembles its effects on L1 retrotransposition in two regards, first, that hA3G showed similar levels of inhibitory activity against the both retrotransposition events (Figure 1C and ref [37,40]), and second, that the hA3G restriction of retrotransposition is independent of deamination in both cases (Figure 3C and refs. 35,37). These similarities prompted us to determine whether the inhibition of L1 retrotransposition by hA3G requires hA3G oligomerization, as does the inhibition of *Alu* retrotransposition. We performed an L1 retrotransposition assay using all hA3G mutants that we created in this study. As expected, the mutants that do not form oligomers, including NΔ30, NΔ60, NΔ90, NΔ120, NΔ150, C97/100A, 5G(24–28), and 4G(124–127), did not inhibit L1 retrotransposition (Figure 7A, 7B, and 7C), whereas, as observed for *Alu* retrotransposition in Figure 3C, the E259Q deamination mutant had a wild-type level of anti-L1 activity (Figure 7B). Thus, the inhibitory effect of hA3G on *Alu* retrotransposition is associated with hA3G oligomerization but independent of its deaminase activity. We therefore postulate that the inhibitory activities of hA3G against *Alu* and L1 retrotransposition might share common mechanism(s). 

## Discussion

Our present study demonstrated that hA3 family proteins inhibit *Alu* retrotransposition at differential levels, which are very similar to the levels at which these host proteins block L1 retrotransposition. With respect to hA3G, the N-terminal 30 amino acids are important for the anti-*Alu* activity. The ability of hA3G to inhibit *Alu* retrotransposition was independent of its deaminase activity but associated with its oligomerization activity, as previously reported by Hulme et al. [35] and Bulliard et al. [34], respectively. In agreement with these findings, we found that the N-terminal 30 amino acids that are responsible for counteracting *Alu* retrotransposition are required for the oligomerization of this protein. We used structural modeling to identify the specific residues among the N-terminal 30 amino acids that are responsible for the oligomerization of hA3G. We finally identified amino acid residues 24–28 of hA3G as the contributors of oligomerization. 

Importantly, these residues were also critical for the inhibitory activity of L1 retrotransposon, suggesting that this activity might involve the same mechanism as that of *Alu* retrotransposition. This hypothesis makes sense because *Alu* elements do not encode a functional reverse transcriptase or endonuclease, and therefore, they need to hijack the L1-encoded enzymatic machinery for retrotransposition through mechanisms that are currently unclear. It is intriguing to speculate that hA3G might be able to physically block both the *Alu* and L1 retroelements because hA3G is intrinsically an RNA-binding protein that can associate non-specifically with cellular RNAs [48,59,65,69], including those derived from *Alu* retroelements [34,70], or because this protein might directly interact with the L1 ORF2 protein. It is likely that both cases would result in the effective inhibition of *Alu* reverse transcription, and are dependent on the ability of hA3G to form oligomers. In the former case, *Alu* RNA *per se* might help stabilize hA3G oligomer formation, as suggested in Figure S2.

It was somewhat unexpected to find that the N-terminal 30 amino acids of hA3G are required for oligomerization in our study because amino acid positions 124/127 have previously been reported to be important [34,58,59]. Indeed, although only minor effects of either a single R24 or S28 mutation on oligomerization were shown by Huthoff et al. [59] and Bulliard et al. [34] (the former of which was confirmed in Figure S3A), respectively, our study revealed that the previously unappreciated amino acid positions 24–28 among these first 30 residues are responsible for the ability of hA3G to homooligomerize. The dependence of oligomerization on these residues is most likely because not only the amino acids R24 and S28 but also the residues between them are involved in the formation of the interaction interface of an hA3G dimer, as shown in our structural models (Figure 6). This study also reveals that both the amino acid residues 24–28 and 124–127 are equally important for the oligomerization of hA3G. Regarding this point, we assume that the lack or a functional defect of a single interaction interface would be able to totally abolish the protein-protein interaction by leading to the structural destabilization.

Whereas transcriptional repressors such as SRY, SOX2 and methyl-CpG-binding protein 2 have been reported to negatively regulate L1 retrotransposition at the transcriptional levels [71-73], post-transcriptional L1 regulation (apart from that by endogenously encoded small interfering RNAs [74]) like premature polyadenylation and aberrant splicing of its mRNA was also shown to result in a negative influence on L1 expression [75]. In the latter case, retrotransposition-incompetent L1 elements that encode intact ORF2 protein are still able to create DNA double-strand breaks [76] and therefore keep mobilizing *Alu* elements [5,53]. Particularly in such conditions, hA3 proteins would play pivotal roles in the inhibition of *Alu* retrotransposition, putatively through binding to either the ORF2 protein or *Alu* RNA as described above.

It should be noted that the superfamily-1 RNA helicase protein MOV10 (Moloney Leukemia Virus 10; for review, see ref[77].), which is highly conserved across a wide range of species, has recently been reported to inhibit not only infection by several retroviruses, such as HIV-1, simian immunodeficiency virus, murine leukemia virus, and equine infectious anemia virus [78,79], but also the retrotransposition of endogenous retroelements [80-82], exactly as hA3G does. Most importantly, MOV10 was identified to be a protein interacting with hA3G in an RNA-dependent manner [83], suggesting that these two proteins may play mutually supporting roles in restricting exogenous viruses and endogenous retroelements. Further analyses are required to elucidate the precise mechanisms by which hA3 family proteins negatively regulate *Alu* and L1 retrotransposition, possibly in cooperation with other cellular factor(s). 

## Supporting Information

Figure S1 **Inhibitory effect of hA3G deletion mutants on HIV-1 infection was evaluated by cotransfecting 293T cells with hA3G and VSV-G plasmids, together with a luciferase-based Vif (-** **) Env (-) HIV-1 construct, as described by Iwabu et al**. **(J. Biol. Chem., 285: 35350-8, 2010)**.
After 48 h, each viral supernatant was harvested. Normalized supernatants were incubated with 293T cells for additional 48 h. Cells were then lysed and subjected to luciferase assay. The data shown are the mean ± SD of triplicate experiments. RLU: relative light units.
(TIF)

Figure S2 **Cellular RNA contributes to the stabilization of hA3G’s oligomer.** HA-tagged hA3G-WT in the immunoprecipitate as described in Figure 4, with or without RNase A treatment.
(TIF)

Figure S3 **hA3G mutants with individual amino acid substitutions.** (A) Oligomerization assay was performed by IP-Western blot analysis, as described in Figure 4; upper, IP; lower, cell lysates. (B) An *Alu* retrotransposition assay was performed as described in Figure 1. Crystal violet-stained G418^R^ colonies were counted to determine the level of *Alu* retrotransposition. The data shown are the mean ±SD of triplicate experiments. Mo, mock; WT, wild-type hA3G; GFP, GFP only. *P < 0.05, **P < 0.005, *t*-test.
(TIF)

Figure S4 **Inhibitory effect of hA3G oligomerization mutant proteins on HIV-1 infection.** The assay was performed as described in Figure S1. The data shown are the mean ± SD of triplicate experiments. RLU: relative light units.
(TIF)
