# Supplementary figures and images for: APOBEC3G Oligomerization Is Associated with the Inhibition of Both Alu and LINE-1 Retrotransposition
Source: PLoS One. 2013 Dec 19;8(12):e84228. doi: 10.1371/journal.pone.0084228 (PMC3868573; doi:10.1371/journal.pone.0084228)

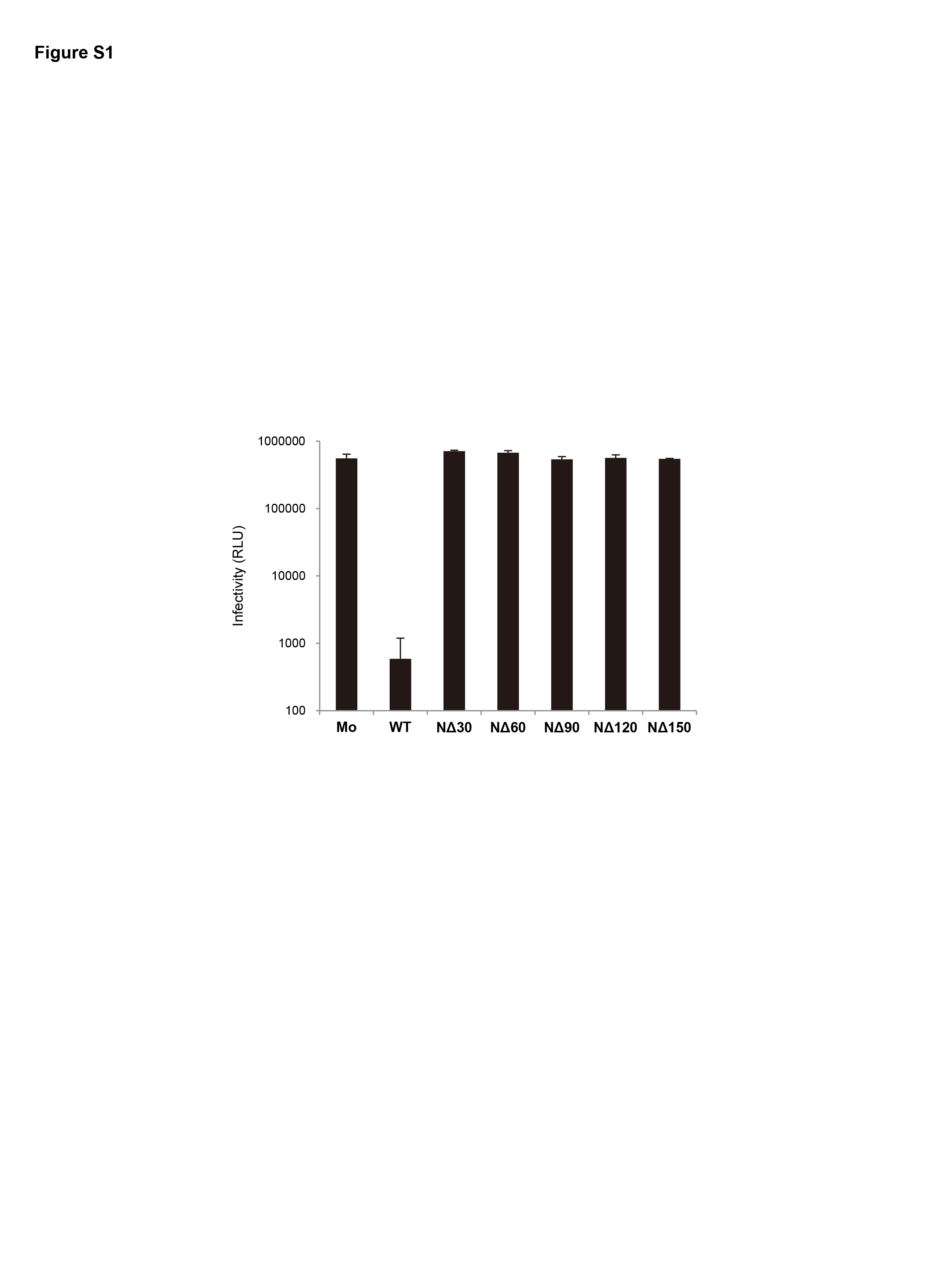

Supplement: Figure S1 — Inhibitory effect of hA3G deletion mutants on HIV-1 infection was evaluated by cotransfecting 293T cells with hA3G and VSV-G plasmids, together with a luciferase-based Vif (- ) Env (-) HIV-1 construct, as described by Iwabu et al. (J. Biol. Chem., 285: 35350-8, 2010). After 48 h, each viral supernatant was harvested. Normalized supernatants were incubated with 293T cells for additional 48 h. Cells were then lysed and subjected to luciferase assay. The data shown are the mean ± SD of triplicate experiments. RLU: relative light units. (TIF) [file pone.0084228.s001.tif]

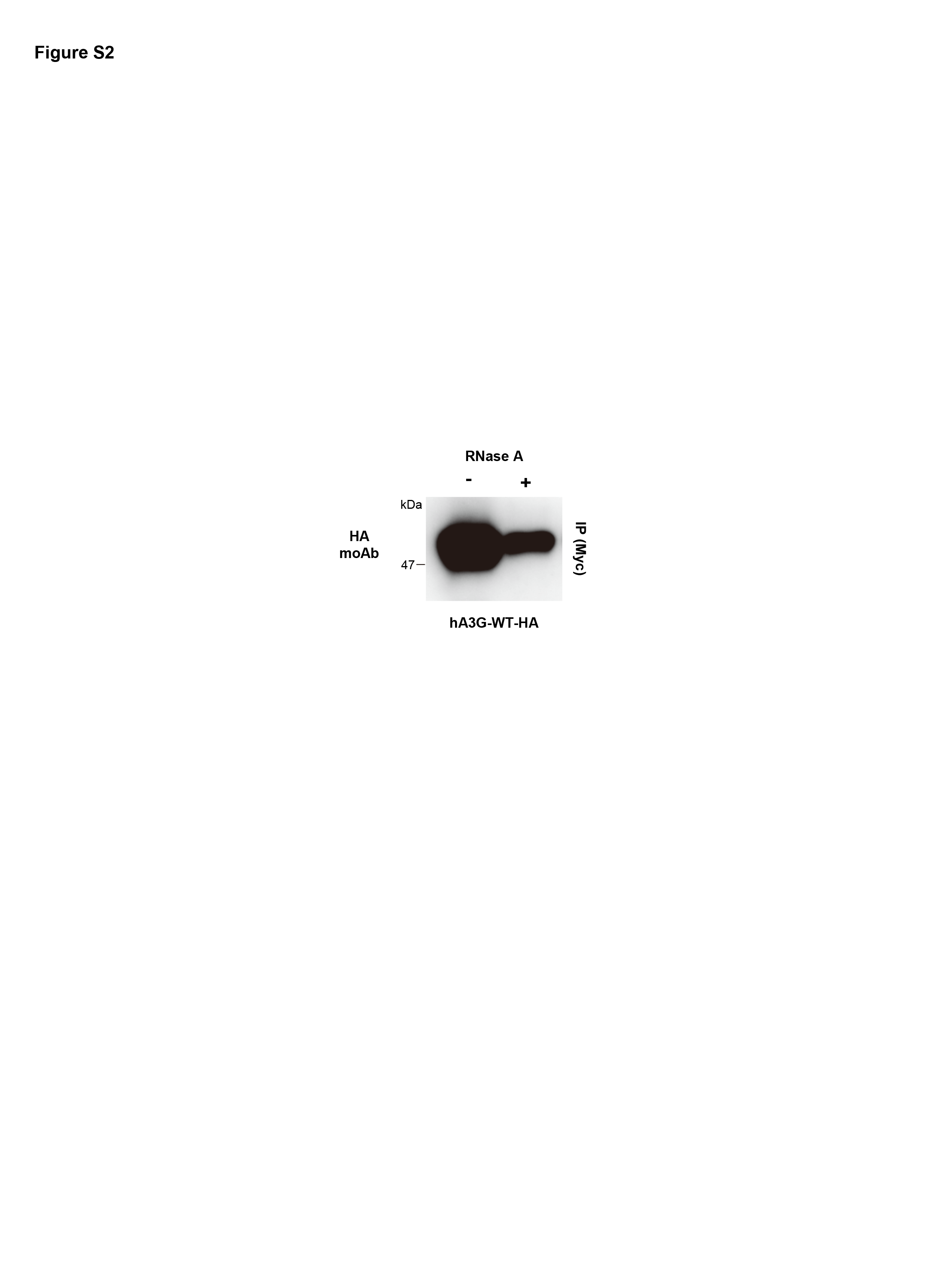

Supplement: Figure S2 — Cellular RNA contributes to the stabilization of hA3G’s oligomer. HA-tagged hA3G-WT in the immunoprecipitate as described in Figure 4, with or without RNase A treatment. (TIF) [file pone.0084228.s002.tif]

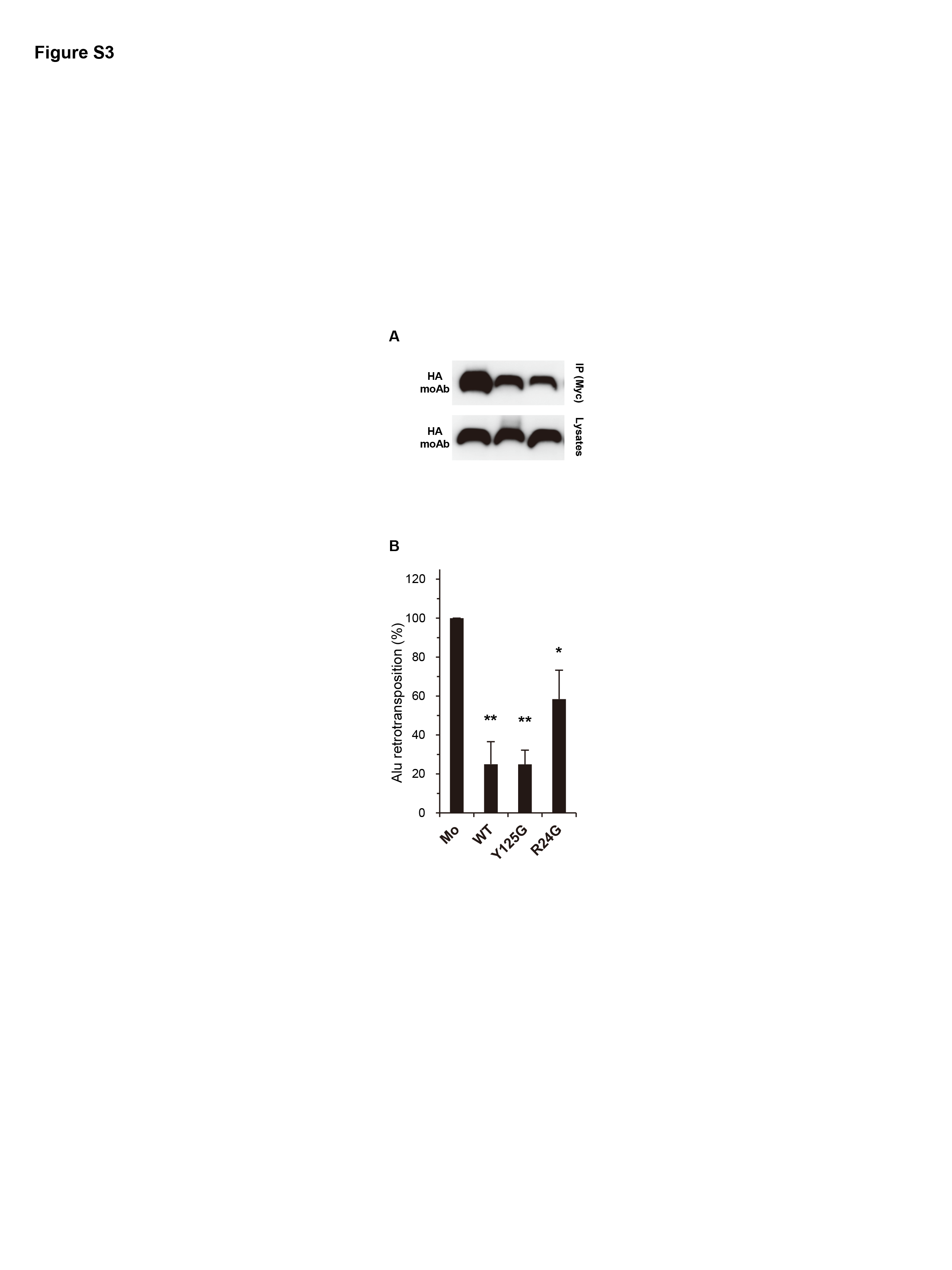

Supplement: Figure S3 — hA3G mutants with individual amino acid substitutions. (A) Oligomerization assay was performed by IP-Western blot analysis, as described in Figure 4; upper, IP; lower, cell lysates. (B) An Alu retrotransposition assay was performed as described in Figure 1. Crystal violet-stained G418R colonies were counted to determine the level of Alu retrotransposition. The data shown are the mean ±SD of triplicate experiments. Mo, mock; WT, wild-type hA3G; GFP, GFP only. *P < 0.05, **P < 0.005, t-test. (TIF) [file pone.0084228.s003.tif]

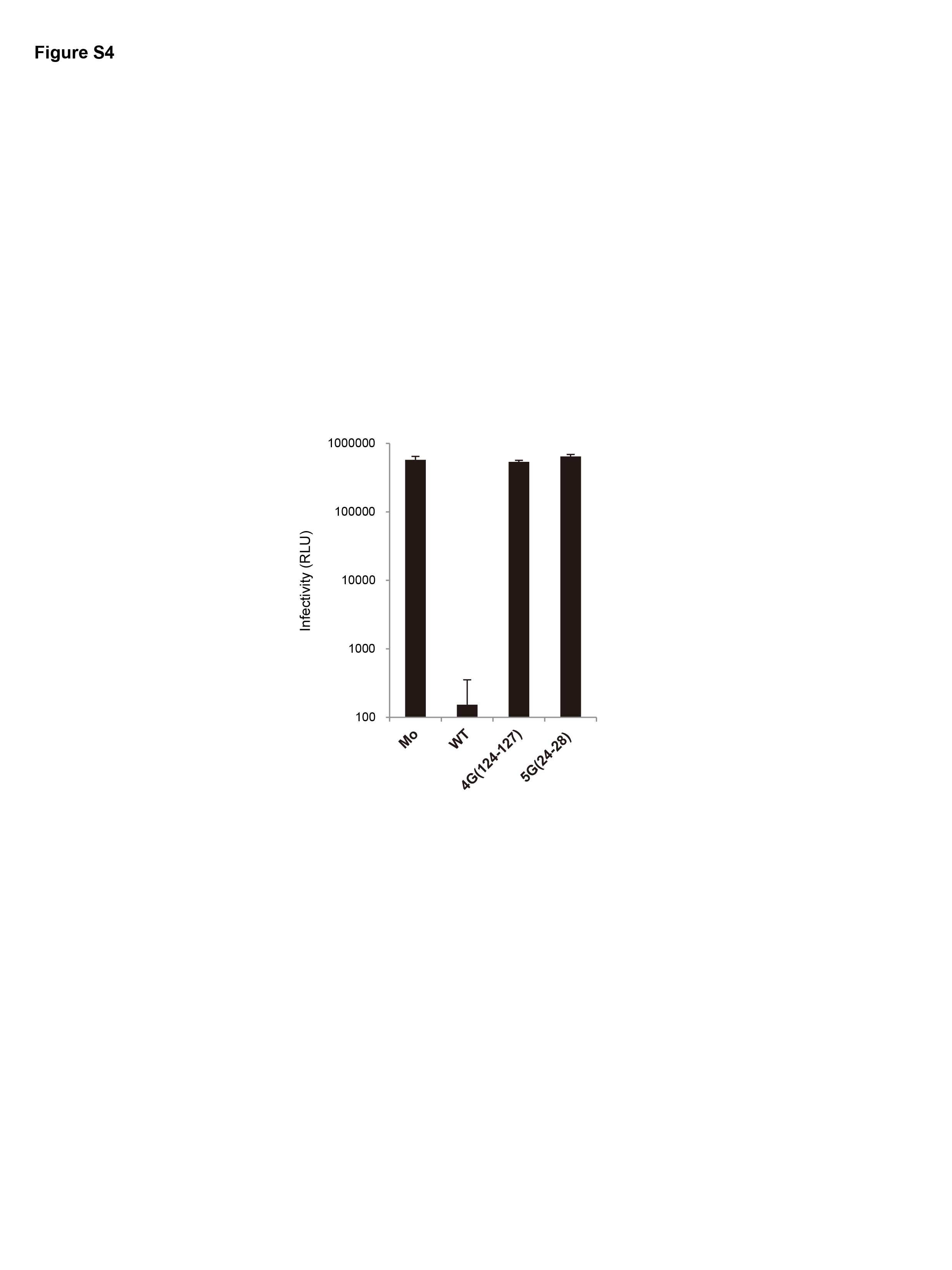

Supplement: Figure S4 — Inhibitory effect of hA3G oligomerization mutant proteins on HIV-1 infection. The assay was performed as described in Figure S1. The data shown are the mean ± SD of triplicate experiments. RLU: relative light units. (TIF) [file pone.0084228.s004.tif]
